# Psycho-Emotional Factors Associated with Internet Gaming Disorder Among Japanese and Israeli University Students and Other Young Adults

**DOI:** 10.3390/bs15070841

**Published:** 2025-06-22

**Authors:** Shai-li Romem Porat, Alexander Reznik, Akihiro Masuyama, Daichi Sugawara, Gal Galya Sternberg, Takahiro Kubo, Richard Isralowitz

**Affiliations:** 1 Regional Alcohol and Drug Abuse Research Center, Ben Gurion University of the Negev, Beer Sheva 84105, Israel; shailir@post.bgu.ac.il (S.-l.R.P.); reznikal@bgu.ac.il (A.R.); galstern@post.bgu.ac.il (G.G.S.); 2 Faculty of Psychology, Iryo Sosei University, Iwaki 971-8550, Fukushima, Japan; masuyama@auecc.aichi-edu.ac.jp; 3 Faculty of Human Services, University of Tsukuba, Tsukuba 305-8577, Ibaraki, Japan; sugawara@human.tsukuba.ac.jp; 4 College of Education, Yokohama National University, Yokohama 240-8501, Kanagawa, Japan; kubo-takahiro-kc@ynu.ac.jp

**Keywords:** gaming disorder, loneliness, burnout, university students, Japan, Israel

## Abstract

Gaming is a popular leisure activity with an increasing number of participants worldwide. It has positive aspects as well as a problematic side—Internet Gaming Disorder (IGD). This behavior attracts concern among mental health and education professionals because of possible negative psycho-emotional factors. This study aimed to assess IGD among Japanese and Israeli university students and other young adults. We explored the association of culture and IGD based on gender, burnout, and loneliness. It was hypothesized that IGD would differentiate based on the respondent country (i.e., Japan or Israel) and gender, with males reporting higher levels. Furthermore, IGD would be associated with higher levels of loneliness and burnout, regardless of country. Qualtrics and Excel platforms were used to collect responses to the Internet Gaming Disorder Scale–Short Form, De Jong Gierveld Loneliness Scale, and Short Burnout Measure (SBM). Data was gathered from a cross-cultural sample of 1318 male and female university students and other young adults in Japan and Israel, between 2022 and 2023. Japanese gamers showed less IGD (*p* < 0.05); and males evidenced higher levels regardless of their country (*p* < 0.001). IGD was significantly associated with loneliness (*p* < 0.001) and burnout (*p* < 0.001). However, multiple regression analysis showed that IGD is predicted only by burnout and gender (*p* < 0.001), Adjusted R^2^ = 0.234. This study provides information for policy, prevention, and intervention purposes targeting burnout particularly among males who are a high-risk group. Additionally, this study contributes to possible joint online program development to reduce IGD among Japanese and Israeli gamers. Further research should examine the association between IGD and loneliness, controlling gender and other factors such as substance use, religiosity, eating behavior, depression, game genre, and motivation to play.

## 1. Introduction

There are more than 2.5 billion gamers worldwide, with an annual market revenue of about $500 billion (Statista, 2024a, 2024b). Gaming behavior promotes critical thinking, collective and individual problem solving, knowledge integration, and experiential learning through feedback mechanisms (Toyoda, 2020; Wideman et al., 2007). However, for some gamers, this activity may be a maladaptive coping mechanism dealing with psycho-emotional conditions (Efrati & Spada, 2023), as evidenced during the COVID-19 pandemic with an increase in gaming disorders (Oka et al., 2021).

The American Psychiatric Association (DSM-5) and the World Health Organization (ICD-11) acknowledge problematic gaming behavior among individuals, particularly adolescents and young adults (H. S. Kim et al., 2022). Based on a systematic review and meta-analysis of gaming disorder across 17 countries, there is a 3.05% prevalence of gaming disorder. This rate tends to be like obsessive–compulsive disorder, but higher than problem gambling (Stevens et al., 2021). Internet Gaming Disorder (IGD) may include emotional distress, depression, impaired cognitive function and social life, and reduced self-esteem among those affected (Benjet et al., 2023; G. Dong & Potenza, 2016; Muyeed et al., 2024; Q. Wang et al., 2019b; Wartberg et al., 2019). The gaming environment is male-dominated (Harvey & Fisher, 2015; Lopez-Fernandez et al., 2019; Martins et al., 2009); and IGD tends to be more prevalent among males (Bonnaire & Baptista, 2019; Liao et al., 2025). As such, we hypothesized that males would report higher levers of IGD. IGD appears worldwide (Stevens et al., 2021); however, there is a dearth of cross-cultural research associated with the issue.

### 1.1. Culture Differences

To understand the impact of IGD from a cross-cultural perspective, we examined Japan, a “collectivist” East Asian culture valuing groups and communities (Berry et al., 1997; Guo, 2022), and Israel, a Middle Eastern country that tends to be individual-oriented (Kasler et al., 2021). These countries differ in many ways, including religion (Central Bureau of Statistics, 2022; Statista, 2024c), spoken and written language, work values (Zwikael et al., 2005), emotions (Aival-Naveh et al., 2022), social norms and expectations (Kurman et al., 2003), self-perception, and communication (Adair & Brett, 2005). Furthermore, Japan and Israel have different security concerns—Japan tends to be prone to natural disasters, including earthquakes and tsunamis (Segev et al., 2022; Toyoda, 2020), while living conditions in Israel are affected by terror attacks and war. (Maoz, 2004; Segev et al., 2022). Owing to the exploratory nature of the current study, we hypothesized that differences in IGD between Japanese and Israeli respondents would be apparent; however, the nature of these differences was not presumed.

### 1.2. Loneliness and Burnout

Loneliness refers to personal feelings about social interactions and relationships perceived to be lacking (Refaeli & Achdut, 2021; Perlman & Peplau, 1981). Research suggests that loneliness can be social and/or emotional (Russell et al., 1984; Weiss, 1975). Emotional loneliness may result from a lack of intimate relationships or meaningful emotional bonds, whereas social loneliness may be caused by an insufficient number of relationships and/or social networks (Gierveld & Tilburg, 2006; Weiss, 1975).

Studies show that increased loneliness is associated with excessive online gaming, problematic video gaming, low self-esteem, high depression, low quality of life, and physical and psychological decline (Lederman, 2024; Ok, 2021; Taniguchi & Kaufman, 2019). Previous studies have attempted to understand loneliness based on cultural differences. It has been found that individuals from collective societies tend to report more loneliness than those from individual-oriented countries (Taniguchi & Kaufman, 2019). As such, it was presumed that Japanese gamers would report higher levels of loneliness; and IGD would be associated with increased loneliness. To the best of our knowledge, the relationship between IGD and loneliness has not been explored in this context. This study aimed to understand this relationship, contributing to an understanding of the association between IGD and cultural factors.

According to B. J. Kim and Choi (2023), burnout is a form of energy depletion. It consists of physical, emotional, or mental exhaustion (Maslach & Jackson, 1981; Malach-Pines, 2005) and is caused by prolonged exposure to stress (Lin et al., 2022). Maladaptive coping strategies are risk factors for burnout (Liu et al., 2020; Rossi et al., 2023). These strategies include addictive behaviors such as excessive Internet use (Tomaszek & Muchacka-Cymerman, 2021) and problematic gaming (Blasi et al., 2019; Schneider et al., 2018). It has been suggested that gaming as a maladaptive coping mechanism is associated with excessive burnout (Tomaszek & Muchacka-Cymerman, 2021); however, only a few studies have assessed the relationship (Cao et al., 2022; Isralowitz et al., 2022a; Lay et al., 2021; Zhang et al., 2022). We hypothesized that IGD is positively associated with burnout cross culturally.

Lastly, limited previous studies have assessed the relationship between these emotional factors and Internet addiction (Gu et al., 2023). As such, assessing burnout and loneliness together in a bifactorial relationship may provide baseline information on how these factors interact.

For this study, we hypothesized that IGD would be differentiated based on the respondent country (i.e., Japan or Israel) and gender with males reporting higher levels. Furthermore, we hypothesized that increased IGD would be associated with higher levels of loneliness and burnout, regardless of country.

## 2. Methods

Internet gaming in the DSM-5 notes that the disorder must cause “significant impairment” in several aspects of a person’s life. The IGD criteria (American Psychiatric Association, 2013) have a lower threshold for identifying problematic gaming than those used by the World Health Organization (2018) (Ko et al., 2020). The APA definition was used for this study.

Survey respondents were from the Aichi University of Education, University of Tsukuba, Yokohama National University (Japan), and the Ben Gurion University of the Negev (Israel). The research activities involving human participants were compliant with the ethical guidelines of the universities involved. The study protocol was approved by the Ethics Committee of the Spitzer Department of Social Work, Ben Gurion University of the Negev (Approval Number: 12062022).

### Participants

Invitations to participate in the survey were posted on university media and online in gaming chats and groups. An online link was provided, along with contact information, if necessary, for additional survey details. Participants were informed of ethical standards and confidentiality of their responses. Their responses integrated consent to participate in the study. Data was collected between 2022 and 2023 from university students and other young adults who voluntarily participated in the study. The study cohort included 1318 Japanese (*n* = 602) and Israeli (*n* = 716) students and other young adults, 68.8% male, 29.9% female, and 1.3% other, who mentioned engaging in any form of video or Internet gaming, online or offline, during the past year. The sample size calculation for the case of cross-sectional studies was carried out in accordance with sample size calculation in medical studies (Charan et al., 2021), and the prevalence of online gaming disorder among university students and young adults (Ohayon & Roberts, 2021; Oka et al., 2021). Respondents who did not complete the study (up to 3%) were automatically excluded from analysis, including those who answered negatively to the question about using online games over the past 12 months or those who reported playing 0 h a week.

Table 1 shows respondent background characteristics.

## 3. Measures

The Qualtrics and Excel platforms were used to respond to the nine-item Internet Gaming Disorder Scale–Short Form (IGDS9-SF) (Pontes & Griffiths, 2015). This was the first brief standardized psychometric tool to evaluate IGD, in coherence with the criteria of the American Psychiatric Association in the 5th edition of the Diagnostic and Statistical Manual of Mental Disorders. The IGDS9-SF statement agreement levels were evaluated using a 5-point Likert scale from 1 (Never) to 5 (Very Often). Higher scores indicate higher levels of IGD.

Other instruments used for the survey were the six-item De Jong Gierveld Loneliness Scale (SLS) (Gierveld & Tilburg, 2006) to gauge emotional, social, and general loneliness and the ten-item Short Burnout Measure (SBM) (Malach-Pines, 2005). In both scales, higher scores indicate stronger appearance of the phenomena.

The choice of instruments used in the survey was determined by the analysis of other studies on gaming disorder and the instruments they used (mainly IGDS9-SF). Concern about the problem of gaming disorder has increased during the COVID-19 pandemic and its impact on mental health and psycho-emotional well-being. This is related to the choice of such instruments as the loneliness scale (six-item De Jong Gierveld Loneliness Scale) and burnout (ten-item Short Burnout Measure).

The survey instruments were translated from English to Hebrew and Japanese and back to English by native speakers of both languages to ensure that the content and vocabulary were appropriate for the population surveyed. The instruments showed good internal consistency, as evidenced by Cronbach’s alpha scores (IGDS9-SF = 0.848, SLS = 0.687, and SBM = 0.914). The data was analyzed using SPSS version 25. The methods employed in the analysis included multiple regression, Pearson’s chi-squared test for dichotomous variables, with phi (φ) or Cramer’s V calculations, the Mann–Whitney test with r, t test, Cohen’s d, Kruskal–Wallis test and one- and two-way ANOVA for continuous variables along with eta squared (η^2^).

## 4. Results

The average number of weekly hours spent playing games was 12.8 (SD = 13.4). Based on the total study cohort, males spent more time gaming than females (t(1197) = 8.730, *p* < 0.001, d = 0.543), and country status showed that Japanese students were less involved with gaming than those from Israel (t(1214) = 17.772, *p* < 0.001, d = 1.019 (large effect)).

The average score on the Internet Gaming Disorder Scale–Short Form (IGDS9-SF) was 18.7 (SD = 6.6), with a range of 9 to 45. The mean IGDS9-SF scores were 18.3 (SD = 7.2) and 19.1 (SD = 5.9) for the Japanese and Israeli participants, respectively (t(1200) = 2.131; *p* < 0.05, d = 0.129). As for the first hypothesis regarding country and gender, significant IGD differences were found based on gender—male more than female (t(1183) = 6.324; *p* < 0.001, d = 0.394 (small effect)).

**Figure 1:**
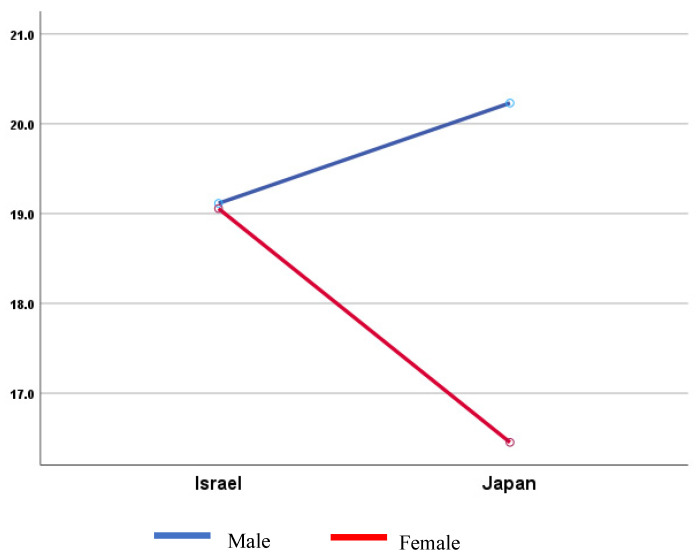
IGDS9-SF scores by country and gender.

Two-way ANOVA shows a significant difference in IGDS9-SF scores based on country and gender (F(1,1181) = 14.751; *p* < 0.001; η^2^ = 0.012 (small effect)) (Figure 1).

**Figure 2:**
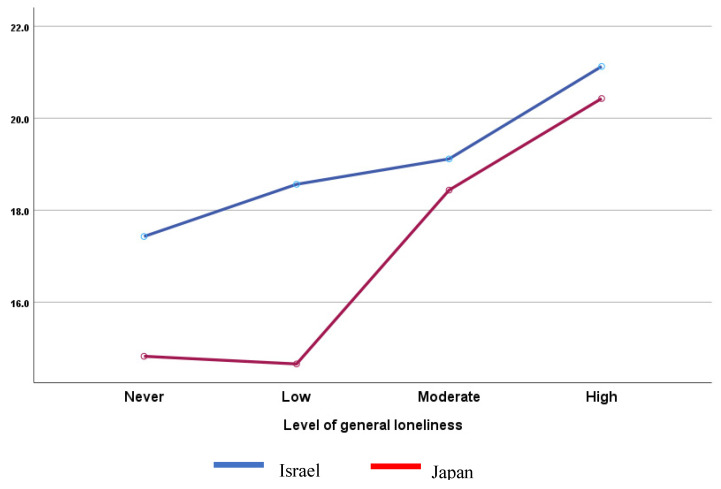
IGDS9-SF scores by country and loneliness.

Regarding the De Jong Gierveld Loneliness Scale (SLS), Japanese students reported more emotional, social and general loneliness than those from Israel (Z = −4.860, *p* < 0.001, r = 0.15 (small effect); Z = −5.737, *p* < 0.001, r = 0.17 (small effect); Z = −6.462, *p* < 0.001, r = 0.20 (small effect), respectively). In addition, females report more general loneliness than males (Z = −2.198, *p* < 0.05, r = 0.07 (small effect)). As for the second hypothesis, A high level of loneliness was associated with a high level of IGD (F(3,1072) = 21.656, *p* < 0.001, η^2^ = 0.057 (medium effect)). A two-way ANOVA shows a significant difference in IGDS9-SF scores based on country and loneliness status (F(3,1068) = 4.186, *p* < 0.01, η^2^ = 0.012 (small effect)) (Figure 2).

The average respondent burnout score was 24.8 (SD = 8.8), with a range of 10 to 50. The mean SBM scores were 25.5 (SD = 9.0) and 24.0 (SD = 8.4) for Japanese and Israeli, respectively (t(1105) = 2.730; *p* < 0.01, d = 0.165 (small effect)). Significant differences in burnout were found based on gender status, female more than male (t(1090) = 3.278; *p* < 0.001, d = 0.210 (small effect)).

For further analysis, the burnout scale results were divided into three categories: low (the first 25% of results), medium (the next 50%), and high (the remaining 25%). To examine the last hypothesis, a one-way ANOVA was calculated, showing a significant difference in IGDS9-SF scores based on burnout level; higher burnout was associated with higher IGDS9-SF scores (F(2,1090) = 96.325, *p* < 0.001, η^2^ = 0.150 (large effect)). Regardless of country, two-way ANOVA shows a significant difference in IGDS9-SF scores based on gender and burnout level (F(2,1072) = 5.692, *p* < 0.01, η^2^ = 0.011 (small effect)).

The Kruskal–Wallis test shows significant differences in the levels of emotional, social, and general loneliness based on burnout—higher burnout levels were associated with more emotional, social, and general loneliness (H = 238.7, *p* < 0.001, η^2^ = 0.22 (large effect); H = 85.6, *p* < 0.001, η^2^ = 0.08 (moderate effect); H = 237.9, *p* < 0.001, η^2^ = 0.22 (large effect), respectively).

Table 2 shows the multiple regression analysis results for IGDS9-SF scores. The proportion of variation (i.e., Adjusted R^2^) for IGD predicted by these variables was 0.237. Additional independent variables, such as country and loneliness, did not significantly increase the proportion of the explained variance.

## 5. Discussion and Conclusions

This study assessed loneliness and burnout factors associated with IGD among university students and other young adults in Japan and Israel. The first study hypothesis was confirmed, country status is associated with IGD—Japanese gamers have significantly lower levels of IGD than those from Israel, and females less than males. Regarding culture, another study examining IGD found opposite results. Specifically, Italian gamers (individualistic culture) showed more severe IGD than those from Peru (more collectivist culture) (Varchetta et al., 2024). This finding may be explained by the fact that Italy (a European country) and Peru (a South American country) are culturally different than Japan and Israel, besides having similar cultural orientations (i.e., individual/collective). The higher levels of loneliness and burnout found among the Japanese cohort with lower IGD levels indicate the need to examine additional factors. Regarding gender, present findings are consistent with other studies that show higher levels of IGD among males (Bonnaire & Baptista, 2019; G.-H. Dong & Potenza, 2022; Konstantinov et al., 2024; M. Wang et al., 2019a; Zhou et al., 2021).

The second hypothesis regarding loneliness was confirmed, Japanese gamers report more loneliness. This result is consistent with studies that compared loneliness among Japanese and Israeli high school (Hosozawa et al., 2024) and university students (Isralowitz et al., 2022b). However, this association may be affected by time-period and living conditions. For example, during the COVID-19 pandemic, Japanese adults reported less loneliness than those from Israel (Lederman, 2023).

Present findings confirmed that IGD is positively associated with burnout and loneliness (emotional and social). This outcome is consistent with other studies that have found problematic gaming to be linked to exhaustion, burnout (Cao et al., 2022; Lay et al., 2021; Zhang et al., 2022), and loneliness (Cudo et al., 2022; Hosozawa et al., 2024; Li et al., 2021; Niazi et al., 2024; Pallavicini et al., 2022; Tras, 2019; Vuorinen et al., 2024; Yu et al., 2022, 2023).

Moreover, country and loneliness did not predict IGD. Although IGD levels differ between Japanese and Israeli gamers, these differences are small compared with gender and other significant factors. This may explain why gender is a predictor of IGD, while country is not. Previous studies showed that loneliness and IGD differ based on gender status (Cudo et al., 2019). Present findings show higher loneliness levels among females; however, they spent less time playing games and had less IGD. The inclusion of male and female gamers in the study cohort may explain why loneliness was not a significant predictor of IGD. Another explanation may be rooted in the effect sizes. Although statistically significant associations were found between IGD and loneliness as well as between loneliness, country, and gender, the effect sizes were small. This may explain why loneliness was not a significant predictor of IGD in this study.

The gender and burnout association with IGD has possible significance for prevention and treatment purposes, as well as the development of training and education methods of “help” and education professionals. Further research is needed to understand the association of eating behavior, substance use, and other psycho-emotional factors, such as anxiety and depression, with IGD across countries and over time. In addition, future research should consider factors related to gaming, such as differentiating game genres (e.g., First Person Shooter [FPS], Massive Multiplayer Online Role-Playing Games [MMORPG], and Real Time Strategy [RTS]) or the gamer’s motivations to play. Among the possible questions for additional research are: Is the association between IGD and substance use differentiate between Japan and Israel; Does gaming motivation explain the association of loneliness, burnout and IGD; and do Gaming motivations differentiate between individual-oriented and collectivist countries.

## 6. Limitation

Responses were not randomly sampled. The student and young adult responses were drawn from several universities and online gaming groups (e.g., “Gamers in Israel”—Facebook Group), which may not be representative. Additionally, a bias is possible due to the dominance of male students in the Israeli sample, and the self-report nature of the data. Furthermore, the study did not measure other psycho-emotional factors such as depression, which may be linked to IGD, and did not differentiate between gaming genres and motivations to play.

## Figures and Tables

**Table 1:** Demographic characteristics.

	Total (n = 1318)	Japan (n = 602)	Israel (n = 716)	*p* Value	Cohen’s d or Cramer’s V
Age,					6.211
Mean (SD)	25.8 (6.7)	23.2 (4.7)	28.1 (7.2)	<0.001	
Median	24.0	22.0	27.0		
Gender, n (%)					0.436
Male	907 (68.8)	282 (46.8)	625 (87.3)	<0.001	
Female	394 (29.9)	305 (50.7)	89 (12.4)		
Other ^1^	17 (1.3)	15 (2.5)	2 (0.3)		

^1^ In further analyses based on gender, the category ‘other’ is not used.

**Table 2:** IGDS9-SF scores—multiple regression result.

Variable	*B*	95% CI for *B*		SE *B*	β
		*LL*	*UL*		
Constant	17.326	14.559	20.094	1.410	
Burnout	0.315	0.269	0.361	0.024	0.414 ***
Gender	−3.365	−4.180	−2.550	0.415	−0.241 ***
Age	−0.069	−0.133	−0.006	0.032	−0.065 *

Note. CI = confidence interval; *LL* = lower limit; *UL* = upper limit. * *p* < 0.05; *** *p* < 0.001.

## Data Availability

The data presented in this study are available on request from the corresponding author. The data are not publicly available due to privacy.

## References

[B1-behavsci-15-00841] Adair W. L., Brett J. M. (2005). The negotiation dance: Time, culture, and behavioral sequences in negotiation. Organization Science.

[B2-behavsci-15-00841] Aival-Naveh E., Rothschild-Yakar L., Park J., Kurman J. (2022). The value of thinking about feelings across cultures: A preliminary investigation of the mentalizing values scale. Journal of Cross-Cultural Psychology.

[B3-behavsci-15-00841] American Psychiatric Association (2013). Diagnostic and statistical manual of mental disorders.

[B4-behavsci-15-00841] Benjet C., Orozco R., Albor Y. C., Contreras E. V., Monroy-Velasco I. R., Hernández Uribe P. C., Báez Mansur P. M., Covarrubias Díaz Couder M. A., Quevedo Chávez G. E., Gutierrez-García R. A., Machado N., Andersson C., Borges G. (2023). A longitudinal study on the impact of Internet gaming disorder on self-perceived health, academic performance, and social life of first-year college students. The American Journal on Addictions.

[B5-behavsci-15-00841] Berry J. W., Poortinga Y. H., Pandey J. (1997). Handbook of cross-cultural psychology: Social behavior and applications.

[B6-behavsci-15-00841] Blasi M. D., Giardina A., Giordano C., Coco G. L., Tosto C., Billieux J., Schimmenti A. (2019). Problematic video game use as an emotional coping strategy: Evidence from a sample of MMORPG gamers. Journal of Behavioral Addictions.

[B7-behavsci-15-00841] Bonnaire C., Baptista D. (2019). Internet gaming disorder in male and female young adults: The role of alexithymia, depression, anxiety and gaming type. Psychiatry Research.

[B8-behavsci-15-00841] Cao H., Zhang K., Ye D., Cai Y., Cao B., Chen Y., Hu T., Chen D., Li L., Wu S., Zou H., Wang Z., Yang X. (2022). Relationships between job stress, psychological adaptation and internet gaming disorder among migrant factory workers in China: The mediation role of negative affective states. Frontiers in Psychology.

[B9-behavsci-15-00841] Central Bureau of Statistics (2022). The social survey.

[B10-behavsci-15-00841] Charan J., Kaur R., Bhardwaj P., Singh K., Ambwani S. R., Misra S. (2021). Sample size calculation in medical research: A primer. Annals of the National Academy of Medical Sciences (India).

[B11-behavsci-15-00841] Cudo A., Kopiś N., Zabielska-Mendyk E. (2019). Personal distress as a mediator between self-esteem, self-efficacy, loneliness and problematic video gaming in female and male emerging adult gamers. PLoS ONE.

[B12-behavsci-15-00841] Cudo A., Wojtasiński M., Tużnik P., Fudali-Czyż A., Griffiths M. D. (2022). The relationship between depressive symptoms, loneliness, self-control, and gaming disorder among polish male and female gamers: The indirect effects of gaming motives. International Journal of Environmental Research and Public Health.

[B13-behavsci-15-00841] Dong G., Potenza M. N. (2016). Risk-taking and risky decision-making in Internet gaming disorder: Implications regarding online gaming in the setting of negative consequences. Journal of Psychiatric Research.

[B14-behavsci-15-00841] Dong G.-H., Potenza M. N. (2022). Considering gender differences in the study and treatment of internet gaming disorder. Journal of Psychiatric Research.

[B15-behavsci-15-00841] Efrati Y., Spada M. M. (2023). “I have no control over how much time I play” the metacognitions about online gaming scale: Evidence from a cross-cultural validation among Israeli adolescents. Addictive Behaviors.

[B16-behavsci-15-00841] Gierveld J. D. J., Tilburg T. V. (2006). A 6-item scale for overall, emotional, and social loneliness: Confirmatory tests on survey data. Research on Aging.

[B17-behavsci-15-00841] Gu J., Wu P., Luo Y., He X., Fu L., Liu H., Lin F., Xu Q., Wu X. (2023). Internet addiction, loneliness, and academic burnout among Chinese college students: A mediation model. Frontiers in Psychiatry.

[B18-behavsci-15-00841] Guo Z. (2022). A review of social and cultural causes of Hikikomori: Collectivism in Japan. Advances in Social Science, Education and Humanities Research.

[B19-behavsci-15-00841] Harvey A., Fisher S. (2015). “Everyone Can Make Games!”: The post-feminist context of women in digital game production. Feminist Media Studies.

[B20-behavsci-15-00841] Hosozawa M., Igami K., Stanyon D., Charvat H., Knowles G., Nishida A., Shimizu T., Ikeda A., Iso H. (2024). Global trends in adolescent loneliness from 2000 to 2018: A cross-national investigation of 1.6 million students from 79 countries *(SSRN Scholarly Paper 4754870)*.

[B21-behavsci-15-00841] Isralowitz R., Romem Porat S., Zolotov Y., Yehudai M., Dagan A., Reznik A. (2022a). Gaming disorder and psycho-emotional wellbeing among male university students and other young adults in Israel. International Journal of Environmental Research and Public Health.

[B22-behavsci-15-00841] Isralowitz R., Yehudai M., Sugawara D., Masuyama A., Romem Porat S., Dagan A., Reznik A. (2022b). Economic impact on health and well-being: Comparative study of Israeli and Japanese university “help” profession students. Social Sciences.

[B23-behavsci-15-00841] Kasler J., Zysberg L., Gal R. (2021). Culture, collectivism-individualism and college student plagiarism. Ethics & Behavior.

[B24-behavsci-15-00841] Kim B. J., Choi C. J. W. (2023). Impact of compensation and willingness to keep same career path on burnout among long-term care workers in Japan. Human Resources for Health.

[B25-behavsci-15-00841] Kim H. S., Son G., Roh E.-B., Ahn W.-Y., Kim J., Shin S.-H., Chey J., Choi K.-H. (2022). Prevalence of gaming disorder: A meta-analysis. Addictive Behaviors.

[B26-behavsci-15-00841] Ko C.-H., Lin H.-C., Lin P.-C., Yen J.-Y. (2020). Validity, functional impairment and complications related to Internet gaming disorder in the DSM-5 and gaming disorder in the ICD-11. Australian & New Zealand Journal of Psychiatry.

[B27-behavsci-15-00841] Konstantinov V., Mynbayeva A., Gritsenko V., Stelmakh S., Reznik A., Porat S. R., Isralowitz R. (2024). Gaming disorder among Russian and Kazakh university students. Discover Global Society.

[B28-behavsci-15-00841] Kurman J., Yoshihara-Tanaka C., Elkoshi T. (2003). Is self-enhancement negatively related to constructive self-criticism? Self-enhancement and self-Criticism in Israel and in Japan. Journal of Cross-Cultural Psychology.

[B29-behavsci-15-00841] Lay J. P. A., Puspitawati T., Rodiyah (2021). The relationship between playing online games and burnout among students in Yogyakarta. The International Conference on Public Health Proceeding.

[B30-behavsci-15-00841] Lederman Z. (2023). Loneliness at the age of COVID-19. Journal of Medical Ethics.

[B31-behavsci-15-00841] Lederman Z. (2024). Against loneliness we unite: A solidarity-based account of loneliness. Bioethics.

[B32-behavsci-15-00841] Li L., Niu Z., Griffiths M. D., Wang W., Chang C., Mei S. (2021). A network perspective on the relationship between gaming disorder, depression, alexithymia, boredom, and loneliness among a sample of Chinese university students. Technology in Society.

[B33-behavsci-15-00841] Liao Z., Le J., Chen X., Tang Y., Shen H., Huang Q. (2025). Gender differences in problematic gaming among Chinese adolescents and young adults. BMC Psychiatry.

[B34-behavsci-15-00841] Lin H., Li Z., Yan M. (2022). Burn-out, emotional labour and psychological resilience among gastroenterology nurses during COVID-19: A cross-sectional study. BMJ Open.

[B35-behavsci-15-00841] Liu X., Chen J., Wang D., Li X., Wang E., Jin Y., Ma Y., Yu C., Luo C., Zhang L., Liu C., Zhou Y., Yang L., Song J., Bai T., Hou X. (2020). COVID-19 outbreak can change the job burnout in health care professionals. Frontiers in Psychiatry.

[B36-behavsci-15-00841] Lopez-Fernandez O., Williams A. J., Griffiths M. D., Kuss D. J. (2019). Female gaming, gaming addiction, and the role of women within gaming culture: A narrative literature review. Frontiers in Psychiatry.

[B37-behavsci-15-00841] Malach-Pines A. (2005). The burnout measure, short version. International Journal of Stress Management.

[B38-behavsci-15-00841] Maoz Z. (2004). Pacifism and fightaholism in international politics: A structural history of national and dyadic conflict, 1816–1992. International Studies Review.

[B39-behavsci-15-00841] Martins N., Williams D. C., Harrison K., Ratan R. A. (2009). A content analysis of female body imagery in video games. Sex Roles.

[B40-behavsci-15-00841] Maslach C., Jackson S. E. (1981). The measurement of experienced burnout. Journal of Organizational Behavior.

[B41-behavsci-15-00841] Muyeed A., Talukder A., Rahman R., Rumi M. H. (2024). Assessing the impact of emotional distress on internet gaming disorder among youth in Bangladesh: A cross-sectional survey. Mental Health and Social Inclusion.

[B42-behavsci-15-00841] Niazi A., Gul M., Niazi Y. (2024). The association between loneliness, social anxiety, and gaming addiction in male university students. Bulletin of Business and Economics (BBE).

[B43-behavsci-15-00841] Ohayon M. M., Roberts L. (2021). Internet gaming disorder and comorbidities among campus-dwelling U.S. university students. Psychiatry Research.

[B44-behavsci-15-00841] Ok C. (2021). Extraversion, loneliness, and problematic game use: A longitudinal study. Personality and Individual Differences.

[B45-behavsci-15-00841] Oka T., Hamamura T., Miyake Y., Kobayashi N., Honjo M., Kawato M., Kubo T., Chiba T. (2021). Prevalence and risk factors of internet gaming disorder and problematic internet use before and during the COVID-19 pandemic: A large online survey of Japanese adults. Journal of Psychiatric Research.

[B46-behavsci-15-00841] Pallavicini F., Pepe A., Mantovani F. (2022). The effects of playing video games on stress, anxiety, depression, loneliness, and gaming disorder during the early stages of the COVID-19 pandemic: PRISMA systematic review. Cyberpsychology, Behavior, and Social Networking.

[B47-behavsci-15-00841] Perlman D., Peplau L. A. (1981). Toward a social psychology of loneliness. Personal Relationships.

[B48-behavsci-15-00841] Pontes H. M., Griffiths M. D. (2015). Measuring DSM-5 internet gaming disorder: Development and validation of a short psychometric scale. Computers in Human Behavior.

[B49-behavsci-15-00841] Refaeli T., Achdut N. (2021). Financial strain and loneliness among young adults during the COVID-19 pandemic: The role of psychosocial resources. Sustainability.

[B50-behavsci-15-00841] Rossi M. F., Gualano M. R., Magnavita N., Moscato U., Santoro P. E., Borrelli I. (2023). Coping with burnout and the impact of the COVID-19 pandemic on workers’ mental health: A systematic review. Frontiers in Psychiatry.

[B51-behavsci-15-00841] Russell D., Cutrona C. E., Rose J., Yurko K. (1984). Social and emotional loneliness: An examination of weiss’s typology of loneliness. Journal of Personality and Social Psychology.

[B52-behavsci-15-00841] Schneider L. A., King D. L., Delfabbro P. H. (2018). Maladaptive coping styles in adolescents with internet gaming disorder symptoms. International Journal of Mental Health and Addiction.

[B53-behavsci-15-00841] Segev E., Tago A., Watanabe K. (2022). Could leaders deflect from political scandals? Cross-national experiments on diversionary action in Israel and Japan. International Interactions.

[B54-behavsci-15-00841] Statista (2024a). Global video game industry revenue 2029.

[B55-behavsci-15-00841] Statista (2024b). Global video game users 2029.

[B56-behavsci-15-00841] Statista (2024c). Japan-Religious affiliation 2021.

[B57-behavsci-15-00841] Stevens M. W., Dorstyn D., Delfabbro P. H., King D. L. (2021). Global prevalence of gaming disorder: A systematic review and meta-analysis. Australian & New Zealand Journal of Psychiatry.

[B58-behavsci-15-00841] Taniguchi H., Kaufman G. (2019). Self-construal, social support, and loneliness in Japan. Applied Research in Quality of Life.

[B59-behavsci-15-00841] Tomaszek K., Muchacka-Cymerman A. (2021). The mediating effect of student school burnout on the relationship between coping strategies and Internet addiction. Current Issues in Personality Psychology.

[B60-behavsci-15-00841] Toyoda Y. (2020). A framework of simulation and gaming for enhancing community resilience against large-scale earthquakes: Application for achievements in Japan. Simulation & Gaming.

[B61-behavsci-15-00841] Tras Z. (2019). Internet addiction and loneliness as predictors of internet gaming disorder in adolescents. Educational Research and Reviews.

[B62-behavsci-15-00841] Varchetta M., Tagliaferri G., Mari E., Quaglieri A., Cricenti C., Martí-Vilar M. (2024). Cross-cultural examination of problematic internet use and associated psychological variables: A comparative study in Italy, Spain, Ecuador, and Peru. Journal of Clinical Medicine.

[B63-behavsci-15-00841] Vuorinen I., Savolainen I., Sirola A., Oksanen A. (2024). The impacts of stress and loneliness on gambling and gaming problems: A nationwide longitudinal study. International Journal of Social Psychiatry.

[B64-behavsci-15-00841] Wang M., Hu Y., Wang Z., Du X., Dong G. (2019a). Sex difference in the effect of Internet gaming disorder on the brain functions: Evidence from resting-state fMRI. Neuroscience Letters.

[B65-behavsci-15-00841] Wang Q., Ren H., Long J., Liu Y., Liu T. (2019b). Research progress and debates on gaming disorder. General Psychiatry.

[B66-behavsci-15-00841] Wartberg L., Kriston L., Zieglmeier M., Lincoln T., Kammerl R. (2019). A longitudinal study on psychosocial causes and consequences of Internet gaming disorder in adolescence. Psychological Medicine.

[B67-behavsci-15-00841] Weiss R. (1975). Loneliness: The experience of emotional and social isolation.

[B68-behavsci-15-00841] Wideman H. H., Owston R. D., Brown C., Kushniruk A., Ho F., Pitts K. C. (2007). Unpacking the potential of educational gaming: A new tool for gaming research. Simulation & Gaming.

[B69-behavsci-15-00841] World Health Organization (2018). The 11th revision of the international classification of diseases (ICD-11).

[B70-behavsci-15-00841] Yu Y., Fong V. W. I., Ng J. H.-Y., Wang Z., Tian X., Lau J. T. F. (2023). The associations between loneliness, hopelessness, and self-control and internet gaming disorder among university students who were men who have sex with men: Cross-sectional mediation study. Journal of Medical Internet Research.

[B71-behavsci-15-00841] Yu Y., Peng L., Mo P. K. H., Yang X., Cai Y., Ma L., She R., Lau J. T. F. (2022). Association between relationship adaptation and Internet gaming disorder among first-year secondary school students in China: Mediation effects via social support and loneliness. Addictive Behaviors.

[B72-behavsci-15-00841] Zhang M. X., Lam L. W., Wu A. M. S. (2022). Recovery experiences protect emotionally exhausted white-collar workers from gaming addiction. International Journal of Environmental Research and Public Health.

[B73-behavsci-15-00841] Zhou W., Zhang Z., Yang B., Zheng H., Du X., Dong G.-H. (2021). Sex difference in neural responses to gaming cues in Internet gaming disorder: Implications for why males are more vulnerable to cue-induced cravings than females. Neuroscience Letters.

[B74-behavsci-15-00841] Zwikael O., Shimizu K., Globerson S. (2005). Cultural differences in project management capabilities: A field study. International Journal of Project Management.

